# Sedentary accumulation patterns and mental health among university teachers: longitudinal evidence from a single-university two-wave cohort

**DOI:** 10.3389/fpsyg.2026.1904275

**Published:** 2026-09-18

**Authors:** Huakai Zhang, Yongchao Huang, Shiguang Wang, Lei Xiu, Yan Wang

**Affiliations:** 1 Medical College, Zhengzhou University of Industrial Technology, Zhengzhou, Henan, China; 2 Henan Province Engineering Technology Research Center of Smart Health and Elderly Care, Zhengzhou, Henan, China; 3 Zhengzhou Key Laboratory of Motor Function Rehabilitation, Zhengzhou, Henan, China

**Keywords:** mean sitting-bout length, mental health, prolonged sitting, sedentary behavior, sitting patterns, university teachers

## Abstract

**Objective:**

To examine whether 12-month changes in sedentary accumulation were associated with mental-health outcomes among university teachers while accounting for concurrent changes in total sitting time.

**Methods:**

Full-time teachers (*n* = 769) completed 7-day activPAL4 monitoring and the World Health Organization–Five Well-Being Index (WHO-5), 10-item Perceived Stress Scale (PSS-10), and Maslach Burnout Inventory–Emotional Exhaustion (MBI-EE) at baseline and 12 months. Changes in mean sitting-bout length (MBL), the proportion of sitting time accumulated in bouts ≥30 min (Prolonged30%), and total sitting time were entered simultaneously in ANCOVA models adjusted for the corresponding baseline outcome, changes in moderate-to-vigorous physical activity and sleep quality, age, sex, and baseline BMI. Model 2 additionally adjusted for T1 levels of the three sedentary exposures.

**Results:**

In Model 2, greater reductions in MBL were associated with higher WHO-5 scores (*β* = −0.236, 95% CI − 0.441 to −0.032; *p* = 0.023) and lower MBI-EE scores (*β* = 0.125, 95% CI 0.034 to 0.217; *p* = 0.007), while greater reductions in Prolonged30% were associated with lower PSS-10 scores (*β* = 0.043, 95% CI 0.006 to 0.079; *p* = 0.022). Reductions in total sitting time were favorably associated with all three outcomes. An interquartile-range reduction in MBL corresponded to a 0.97-point higher WHO-5 score, about one-tenth of the ~10-point contextual reference value; the corresponding 0.44-point lower PSS-10 estimate for an interquartile-range reduction in Prolonged30% was about 16–20% of the 2.2–2.7-point contextual reference range. All semi-partial *f*^2^ values for focal predictors in the primary Model 2 analyses were <0.02.

**Conclusion:**

More favorable changes in sedentary accumulation were associated with better mental-health outcomes, but the estimated incremental effects were small. Because MBL is structurally linked to sitting volume and bout frequency, its associations are better interpreted as reflecting a more frequently interrupted pattern of sitting rather than an isolated effect of bout duration.

## 1 Introduction

Teachers face sustained cognitive demands and emotional labor, and elevated stress, anxiety, and burnout have been repeatedly documented in this workforce (Agyapong et al., 2022; Ozamiz-Etxebarria et al., 2023; Dreer, 2023). Because teacher mental health relates to instructional quality, student outcomes, and retention, identifying actionable behavioral levers is a public-health priority (Dreer, 2023; Gundlach et al., 2024).

Sedentary behavior—defined as any waking behavior characterized by an energy expenditure ≤1.5 metabolic equivalents while in a sitting, reclining, or lying posture—is a modifiable exposure with two distinct dimensions: total volume and accumulation pattern, referring to how sedentary time is distributed across bouts and breaks (Tremblay et al., 2017; Chastin and Granat, 2010). Greater total sitting time has been associated with adverse health outcomes. Recent device-based prospective evidence has linked high sedentary time with increased risks of all-cause mortality and cardiovascular outcomes, although the magnitude of these associations may vary according to the level of moderate-to-vigorous physical activity (Sagelv et al., 2023; Ajufo et al., 2025). Prolonged, uninterrupted sedentary bouts may also be particularly harmful, and a higher proportion of sedentary time accumulated in bouts lasting ≥30 min has been associated with unfavorable metabolic profiles (Diaz et al., 2017; van der Berg et al., 2016). For mental health, “passive” sedentary contexts (e.g., prolonged television viewing) have been associated with higher depression risk (Huang et al., 2020). Recent evidence indicates that interrupting prolonged sitting with brief activity breaks can improve cardiometabolic responses, particularly postprandial glucose regulation, while workplace interventions incorporating environmental support, motivational strategies, or multiple components can effectively reduce occupational sitting time (Wang et al., 2024; Yin et al., 2024). Current guidelines therefore recommend reducing and regularly interrupting sedentary time (Bull et al., 2020); however, longitudinal evidence in university teachers remains limited, particularly studies that compare the relative importance of volume versus pattern in educational settings.

The Job Demands–Resources framework posits that sustained demands under limited recovery lead to exhaustion and burnout (Bakker and Demerouti, 2017; Bakker et al., 2023), whereas micro-recovery opportunities buffer strain (Bennett et al., 2018; Albulescu et al., 2022). Translating this to sedentary accumulation, frequently breaking up sitting may act as brief restorative episodes that support well-being and reduce exhaustion.

Thigh-worn, device-based measures allow joint assessment of volume and pattern, including mean sitting-bout length (MBL) and the proportion of sitting time accumulated in ≥30-min bouts (“Prolonged30%”), alongside total sitting time (Edwardson et al., 2017; O'Brien et al., 2022). The present study assessed well-being, perceived stress, and emotional exhaustion using the World Health Organization–Five Well-Being Index (WHO-5) (Topp et al., 2015; World Health Organization, 2024), the 10-item Perceived Stress Scale (PSS-10) (Harris et al., 2023), and the Maslach Burnout Inventory–Emotional Exhaustion (MBI-EE) (Maslach and Jackson, 1981), respectively.

Accordingly, this two-wave cohort study examined whether changes in sedentary accumulation characteristics were associated with well-being, perceived stress, and emotional exhaustion at follow-up while accounting for concurrent changes in total sitting time. Because accumulation metrics such as MBL are structurally related to sitting volume and bout frequency, we considered sedentary volume and accumulation characteristics as related dimensions of sitting behavior rather than fully independent exposures. The study therefore aimed to characterize how longitudinal changes in these sedentary-behavior features relate to mental-health outcomes and to inform pragmatic micro-break and sit–stand strategies for academic workplaces.

## 2 Materials and methods

### 2.1 Study design and participants

This two-wave longitudinal cohort study recruited full-time university teachers from a single university in Zhengzhou, Henan Province, China. Baseline assessment (T1) was conducted in March 2024, and follow-up assessment (T2) was conducted approximately 12 months later, in March 2025. To minimize seasonal and academic-calendar bias, both waves were scheduled within the same academic term and season window. All potentially eligible full-time teachers were invited through a university-wide recruitment process. Participation was voluntary, and the baseline sample size was determined by the number of eligible volunteers enrolled during the recruitment period rather than by an *a priori* power calculation.

Participants were eligible if they were full-time teaching faculty at the university, were able to complete the 7-day thigh-worn activPAL4 monitoring protocol and self-administered questionnaires, and provided written informed consent. Before enrollment, individuals were excluded if they were administrative staff, student counselors, logistics staff, or laboratory administrators; had a condition that substantially restricted usual standing or walking; or were participating in another research project during the study period. After enrollment, participants who did not complete the T2 assessment because of long-term leave or other reasons were classified as lost to follow-up. Participants who completed part of the study procedures but lacked valid device data or complete questionnaire data at either T1 or T2 were excluded from the final analytic sample.

The study protocol was reviewed and approved by the Ethics Committee of Zhengzhou University of Industrial Technology (protocol code 2024023). All participants provided written informed consent before data collection.

### 2.2 Procedures

The same standardized data-collection procedures were used at T1 and T2, and both waves were conducted by the same trained research team. At each wave, participants received standardized verbal and written instructions regarding the study protocol, device wear, and questionnaire completion. Reminder messages were sent via WeChat during the 7-day monitoring period to encourage adherence to the device-wear protocol. Electronic questionnaires were completed using the same survey platform and in the same order at both waves. After the monitoring period, participants returned the devices, and the research team checked questionnaire submissions and device records for completeness and data quality. Device data were then downloaded and screened according to the predefined validity criteria. Personal identifiers were removed before analysis, and all study data were stored in an encrypted repository accessible only to authorized members of the research team.

### 2.3 Sedentary behavior assessment

Sedentary behavior was assessed using a thigh-worn activPAL4 monitor (PAL Technologies, Glasgow, UK). At each assessment wave, the device was attached to the anterior thigh and worn during waking hours for 7 consecutive days. Participants were instructed to maintain their usual daily routines and to remove the device only during bathing or swimming. A valid monitoring day was defined as at least 10 h of waking wear time. Consistent with published activPAL field protocols, participants were required to provide at least 4 valid days, including at least 1 weekend day, at both T1 and T2 (Hidde et al., 2024; Edwardson et al., 2017).

Device data were processed using the manufacturer software and standard post-processing procedures. Posture-classified data were used to identify sitting/lying, standing, and stepping periods. A sitting bout was defined as an uninterrupted period classified as sitting/lying, and a break in sitting was defined as a transition from sitting/lying to standing or stepping. For each valid day, we derived total sitting time (min/day), the number of sitting bouts per day, and the number of breaks in sitting per day. Mean sitting-bout length (MBL) was calculated as total sitting time divided by the number of sitting bouts, and Prolonged30% was defined as the percentage of total sitting time accumulated in bouts lasting ≥30 min. Daily values were averaged across valid monitoring days at each wave. Change scores were calculated as T2 minus T1 (Δ = T2 − T1) for MBL, Prolonged30%, total sitting time, sitting bouts/day, and breaks/day. Thus, negative values for ΔMBL, ΔProlonged30%, and Δtotal sitting time indicate reductions in the corresponding sedentary-behavior indicator, whereas positive values for Δbouts/day and Δbreaks/day indicate increases in the daily number of sitting bouts or sitting interruptions, respectively.

### 2.4 Mental health outcomes

Mental health outcomes were assessed at both T1 and T2 using three self-administered scales: the World Health Organization–Five Well-Being Index (WHO-5), the 10-item Perceived Stress Scale (PSS-10), and the Emotional Exhaustion subscale of the Maslach Burnout Inventory (MBI-EE).

The WHO-5 was used to assess subjective well-being. It consists of five items, each scored from 0 to 5. The raw total score ranges from 0 to 25 and was multiplied by 4 to obtain a transformed score ranging from 0 to 100, with higher scores indicating better well-being (Topp et al., 2015; World Health Organization, 2024). The Chinese version of the WHO-5 has demonstrated satisfactory internal consistency, factorial validity, construct validity, and concurrent validity in Chinese populations (Fung et al., 2022). Perceived stress was measured using the PSS-10, which includes 10 items and yields a total score ranging from 0 to 40; higher scores indicate greater perceived stress (Harris et al., 2023). The simplified Chinese version of the PSS-10 has demonstrated satisfactory reliability, factorial validity, concurrent validity, and short-term longitudinal measurement invariance (Lu et al., 2017; Jiang et al., 2023). Emotional exhaustion was assessed using the MBI-EE subscale, with total scores ranging from 0 to 54; higher scores indicate greater emotional exhaustion (Maslach and Jackson, 1981). The Chinese version of the MBI has demonstrated acceptable factorial validity and reliability among Chinese teachers (Yuen et al., 2002).

### 2.5 Covariates

Covariates were selected *a priori* based on their potential associations with sedentary behavior and mental health outcomes. Demographic variables included age and sex, which were collected at baseline using standardized questionnaires. Body mass index (BMI) was calculated as body weight in kilograms divided by height in meters squared (kg/m^2^). Height and body weight were measured on site by trained research staff using standardized procedures.

Sleep quality was assessed at both T1 and T2 using the Pittsburgh Sleep Quality Index (PSQI), with higher scores indicating poorer sleep quality (De Moraes et al., 2024; Guo et al., 2016). The Chinese version of the PSQI has demonstrated acceptable internal consistency, test–retest reliability, and validity in Chinese adults (Tsai et al., 2005). Change in sleep quality was calculated as T2 minus T1 (ΔPSQI = T2 − T1). Moderate-to-vigorous physical activity (MVPA) was estimated from activPAL4-derived activity intensity outputs and expressed as min/day. Daily MVPA was averaged across valid monitoring days at each wave, and change in MVPA was calculated as T2 minus T1.

### 2.6 Statistical analysis

Continuous variables were summarized as means and standard deviations (SDs), and categorical variables were summarized as frequencies and percentages. For descriptive purposes, T1 and T2 values and mean changes with their 95% confidence intervals (CIs) were reported for sedentary-behavior indicators, including sitting bouts/day and breaks/day, as well as for mental-health outcomes, MVPA, and PSQI.

We assessed the distributions of continuous variables and T2 − T1 change scores using histograms, normal Q–Q plots, skewness, excess kurtosis, and the Shapiro–Wilk test. Given the large sample size, we interpreted the Shapiro–Wilk results together with the graphical assessments and distributional measures. For estimates of within-participant changes, we assessed the distributions of the T2 − T1 differences. For the ANCOVA models, we examined the distributions of the standardized model residuals. No substantial departures from normality were identified; therefore, parametric analyses were used.

The primary analyses used an analysis of covariance (ANCOVA) framework to examine whether changes in sedentary accumulation patterns were associated with mental-health outcomes at follow-up. Separate models were fitted for WHO-5, PSS-10, and MBI-EE at T2. Two model specifications were evaluated for each outcome. Model 1 retained the original specification and included the corresponding T1 outcome, ΔMBL, ΔProlonged30%, Δtotal sitting time, ΔMVPA, ΔPSQI, age, sex, and baseline BMI. Model 2 additionally included the corresponding baseline sedentary-behavior exposures (T1 MBL, T1 Prolonged30%, and T1 total sitting time) alongside their respective change terms. In both specifications, the three sedentary-behavior change variables were entered simultaneously to estimate their mutually adjusted associations with the outcome. Model 2 was used to evaluate whether the change-score associations were materially altered after accounting for baseline exposure levels.

To characterize the empirical relationships among the change-score exposures, Pearson correlation coefficients were calculated among ΔMBL, ΔProlonged30%, Δtotal sitting time, and ΔMVPA, with two-sided *p* values reported. Pearson correlations were also calculated between each baseline sedentary exposure and its corresponding change score (T1 MBL with ΔMBL, T1 Prolonged30% with ΔProlonged30%, and T1 total sitting time with Δtotal sitting time). For Model 2, unstandardized regression coefficients (*β*), 95% CIs, and two-sided *p* values were reported for the three baseline sedentary-exposure terms. Multicollinearity was further evaluated separately for each primary ANCOVA model using variance inflation factors (VIFs) and condition indices. VIFs were re-derived from the full predictor set for each fitted model using auxiliary ordinary least-squares regressions with an intercept, with each predictor regressed in turn on all remaining predictors. Condition indices were re-derived from the full fitted design matrix, including the intercept. The design-matrix columns were not mean-centered; instead, each column was scaled to unit Euclidean length, singular-value decomposition was applied, and each condition index was calculated as the ratio of the largest singular value to the corresponding singular value. As part of this planned diagnostic re-derivation, predictor-specific VIFs were calculated for every predictor in the complete fitted models. Model-level summaries are reported in Supplementary Table S2, Panel A, and the full predictor-level VIFs are reported in Panel B; the correlation matrix is reported in Supplementary Table S1.

Given the structural relationship among MBL, total sitting time, sitting-bout frequency, and breaks in sitting, we conducted an alternative parameterization using change in breaks in sitting per day (Δbreaks/day) to express sitting accumulation in more directly interpretable interruption-frequency units. Conditional on total sitting time, Δbreaks/day is an algebraic reparameterization of ΔMBL through the underlying bout frequency; accordingly, this analysis was intended to provide an alternative representation of the same sedentary-accumulation structure rather than an independent test or external corroboration of the MBL findings. In this parameterization, MBL was replaced by breaks/day while the other sedentary-behavior terms were retained. For each mental-health outcome, the model included the corresponding T1 outcome, T1 breaks/day, Δbreaks/day, T1 Prolonged30%, ΔProlonged30%, T1 total sitting time, Δtotal sitting time, ΔMVPA, ΔPSQI, age, sex, and baseline BMI. HC3 heteroskedasticity-consistent standard errors were used. To aid interpretation, the Δbreaks/day coefficient was additionally expressed per 5-break/day increase and per one interquartile-range increase. Because sitting bouts/day and breaks/day represent mechanically closely related counts, they were not entered simultaneously in the same regression model. Results of this alternative parameterization are reported in Supplementary Table S3.

For each focal sedentary-behavior predictor, unstandardized regression coefficients (*β*), 95% CIs, and two-sided *p* values were reported. For the baseline-exposure-adjusted specification (Model 2), standardized *β* coefficients and semi-partial *f*^2^ values were additionally reported to quantify the relative magnitude and incremental contribution of each focal predictor. Exposure units were stated explicitly: ΔMBL was expressed per 1 min/bout, ΔProlonged30% per 1 percentage-point change, and Δtotal sitting time per 30 min/day change. To provide more practically interpretable contrasts, Model 2 estimates were additionally expressed for an interquartile-range change in ΔMBL (4.11 min/bout) and ΔProlonged30% (10.20 percentage points), whereas Δtotal sitting time was interpreted using a 30-min/day contrast. Because change scores were calculated as T2 minus T1, more negative values indicated greater reductions in the corresponding sedentary-behavior indicator. Accordingly, for WHO-5, a negative *β* indicates that greater reductions in the sedentary-behavior predictor were associated with higher well-being, whereas for PSS-10 and MBI-EE, a positive *β* indicates that greater reductions were associated with lower perceived stress or emotional exhaustion. HC3 heteroskedasticity-consistent standard errors were used to reduce sensitivity to heteroscedasticity. Effect modification by baseline perceived stress was examined exploratorily for the association between ΔMBL and WHO-5 at T2. In the primary interaction analysis, baseline PSS-10 was modeled as a continuous moderator. Both ΔMBL and baseline PSS-10 were mean-centered before constructing the interaction term, and the model included their centered main effects and interaction term while retaining the same covariate structure as Model 2. Simple slopes for ΔMBL were estimated at the mean and at 1 SD below and above the mean of baseline PSS-10. As a sensitivity analysis, baseline PSS-10 was additionally dichotomized at the sample-specific 75th percentile (PSS-10 ≥ 21 vs. <21). In this sensitivity model, ΔMBL remained mean-centered, and the model retained the same covariate structure and included both the main effect of the high-stress indicator and the ΔMBL × high-stress interaction term. The effect-modification analyses were exploratory and were not prespecified. No other sedentary-behavior-by-baseline-stress interaction analyses were conducted for the other sedentary exposures or mental-health outcomes.

A sensitivity power analysis was conducted to characterize the smallest incremental effect detectable in the baseline-exposure-adjusted ANCOVA models. With *n* = 769, 12 predictors, a two-sided α level of 0.05, and 80% power, the minimum detectable incremental effect size for a single focal predictor was Cohen’s *f*^2^ ≈ 0.0102. Because the study was capable of detecting effects smaller than the conventional benchmark for a small effect (*f*^2^ = 0.02) (Cohen, 1992), statistical significance was not interpreted as evidence of practical importance; emphasis was instead placed on effect estimates, confidence intervals, standardized coefficients, and semi-partial *f*^2^ values.

Analyses were conducted using the complete-case analytic sample of participants with valid paired sedentary-behavior and mental-health data at both waves. Statistical significance was set at a two-sided α level of 0.05. All analyses were performed using R version 4.5.1.

## 3 Results

### 3.1 Participant characteristics and two-wave changes

Of the 1,134 teachers recruited at baseline, 52 were lost to follow-up, 215 were excluded because of invalid device data, and 98 were excluded because of incomplete questionnaire data. The final analytic sample comprised 769 full-time university teachers with valid paired sedentary-behavior and mental-health data. The mean age of participants was 40.7 years, 51.5% were men, and the mean BMI was 23.5 kg/m^2^ (Table 1).

**Table 1:** Baseline characteristics and two-wave measurements of the analytic sample (*n* = 769).

Variable	T1, mean (SD) or *n* (%)	T2, mean (SD)	Change, mean (SD)	95% CI for change
Sedentary behavior				
MBL, min/bout	20.2 (5.1)	19.6 (6.0)	−0.6 (3.1)	−0.86 to −0.42
Prolonged30%, pp	50.3 (9.7)	47.9 (12.2)	−2.4 (7.7)	−2.96 to −1.86
Total sitting time, min/day	498.7 (89.5)	477.3 (105.5)	−21.4 (54.5)	−25.23 to −17.52
Sitting bouts, n/day	26.7 (11.0)	28.2 (13.8)	1.5 (7.0)	1.00 to 2.00
Breaks in sitting, n/day	25.9 (11.0)	27.3 (13.8)	1.4 (7.0)	0.90 to 1.90
Mental health outcomes				
WHO-5, 0–100	55.8 (16.3)	57.3 (18.8)	1.5 (9.1)	0.84 to 2.14
PSS-10, 0–40	17.3 (6.1)	18.1 (7.3)	0.8 (4.0)	0.50 to 1.07
MBI-EE, 0–54	21.0 (9.1)	21.8 (9.7)	0.8 (4.0)	0.50 to 1.06
Covariates				
MVPA, min/day	34.9 (17.1)	39.1 (23.8)	4.2 (17.5)	2.97 to 5.44
PSQI, 0–21	6.2 (2.7)	5.9 (3.1)	−0.3 (1.6)	−0.40 to −0.18
Age, years	40.7 (8.3)	—	—	—
Male, *n* (%)	396 (51.5)	—	—	—
BMI, kg/m^2^	23.5 (3.5)	—	—	—

Values are means (SDs) unless otherwise indicated. Change was calculated as T2 minus T1. Prolonged30% refers to the percentage of total sitting time accumulated in sitting bouts lasting ≥30 min and is expressed in percentage points (pp). A break in sitting was defined as a transition from sitting/lying to standing or stepping. Negative changes in MBL, Prolonged30%, and total sitting time indicate reductions in sedentary accumulation. A lower PSQI score indicates better sleep quality.

BMI, body mass index; CI, confidence interval; MBL, mean sitting-bout length; MBI-EE, Maslach Burnout Inventory–Emotional Exhaustion; MVPA, moderate-to-vigorous physical activity; PSQI, Pittsburgh Sleep Quality Index; PSS-10, 10-item Perceived Stress Scale; WHO-5, World Health Organization–Five Well-Being Index.

Across the two assessment waves, MBL, Prolonged30%, and total sitting time decreased by a mean of 0.6 min/bout, 2.4 percentage points, and 21.4 min/day, respectively. The mean number of sitting bouts increased from 26.7 (SD 11.0) bouts/day at T1 to 28.2 (SD 13.8) bouts/day at T2, corresponding to a mean change of +1.5 bouts/day (95% CI 1.00 to 2.00). Similarly, breaks in sitting increased from 25.9 (SD 11.0) breaks/day at T1 to 27.3 (SD 13.8) breaks/day at T2, with a mean change of +1.4 breaks/day (95% CI 0.90 to 1.90). WHO-5 increased by 1.5 points, whereas PSS-10 and MBI-EE each increased by 0.8 points. MVPA increased by 4.2 min/day, and PSQI decreased by 0.3 points, indicating modestly improved sleep quality (Table 1).

The T2 − T1 change scores showed approximately symmetric distributions, with absolute skewness values ≤0.234 and absolute excess kurtosis values ≤0.416. Visual inspection of the histograms and normal Q–Q plots indicated no substantial departures from normality. The standardized residuals from the six ANCOVA models (three outcomes × two model specifications) were approximately normally distributed, with skewness ranging from −0.088 to 0.068 and excess kurtosis ranging from −0.380 to 0.142; the corresponding Shapiro–Wilk tests were non-significant.

### 3.2 Associations between changes in sedentary accumulation and mental-health outcomes

Two ANCOVA specifications were examined for each mental-health outcome. Model 1 retained the original specification, in which the three sedentary-behavior change variables were entered simultaneously and the models were adjusted for the corresponding T1 outcome, ΔMVPA, ΔPSQI, age, sex, and baseline BMI. Model 2 additionally included T1 MBL, T1 Prolonged30%, and T1 total sitting time alongside their respective change terms. The estimated associations of the sedentary-behavior change variables were materially unchanged after this additional adjustment for baseline exposure levels, with no material attenuation or reversal in direction (Table 2). The baseline sedentary-exposure terms in Model 2 were not statistically significant for any of the three mental-health outcomes (all *p* ≥ 0.05); their unstandardized coefficients, 95% CIs, and *p* values are reported in Supplementary Table S4.

**Table 2:** Associations of changes in sedentary-behavior indicators with mental-health outcomes at follow-up: original and baseline-exposure-adjusted specifications.

Outcome	Δ Predictor	Model 1 *β* (95% CI)	*p*	Model 2 *β* (95% CI)	*p*	Standardized *β* (Model 2)	Semi-partial *f*^2^ (Model 2)
WHO-5	ΔMBL, per 1 min/bout	−0.233 (−0.437, −0.029)	0.025	−0.236 (−0.441, −0.032)	0.023	−0.040	0.0071
	ΔProlonged30%, per 1 pp	0.005 (−0.076, 0.086)	0.899	0.008 (−0.074, 0.090)	0.846	0.003	0.0001
	Δtotal sitting time, per 30 min/day	−0.529 (−0.883, −0.175)	0.003	−0.532 (−0.888, −0.177)	0.003	−0.052	0.0120
PSS-10	ΔMBL, per 1 min/bout	0.070 (−0.018, 0.158)	0.120	0.068 (−0.020, 0.157)	0.131	0.029	0.0029
	ΔProlonged30%, per 1 pp	0.041 (0.004, 0.077)	0.030	0.043 (0.006, 0.079)	0.022	0.045	0.0070
	Δtotal sitting time, per 30 min/day	0.223 (0.067, 0.378)	0.005	0.223 (0.066, 0.379)	0.005	0.056	0.0105
MBI-EE	ΔMBL, per 1 min/bout	0.127 (0.036, 0.217)	0.006	0.125 (0.034, 0.217)	0.007	0.041	0.0100
	ΔProlonged30%, per 1 pp	0.017 (−0.019, 0.054)	0.355	0.020 (−0.017, 0.057)	0.284	0.016	0.0016
	Δtotal sitting time, per 30 min/day	0.254 (0.096, 0.411)	0.002	0.255 (0.098, 0.413)	0.001	0.048	0.0138

Model 1 reproduces the original specification and includes the corresponding T1 outcome, ΔMBL, and ΔProlonged30%, Δtotal sitting time, ΔMVPA, ΔPSQI, age, sex, and baseline BMI. Model 2 additionally includes T1 MBL, T1 Prolonged30%, and T1 total sitting time. The three sedentary-behavior change indicators were entered simultaneously in both models. Unstandardized regression coefficients (*β*), 95% CIs, and p values are based on HC3 heteroskedasticity-consistent standard errors. Standardized *β* and semi-partial *f*^2^ are reported for Model 2. For interpretability, coefficients for Δ total sitting time are scaled per 30-min/day change; ΔMBL is per 1 min/bout and ΔProlonged30% is per 1 percentage point (pp). Δ = T2 − T1; more negative Δ values indicate greater reductions in the corresponding sedentary-behavior indicator. Accordingly, a negative *β* for WHO-5 and a positive *β* for PSS-10 or MBI-EE indicate that greater reductions in the sedentary-behavior predictor are associated with more favorable mental-health outcomes.

BMI, body mass index; CI, confidence interval; HC3, heteroskedasticity-consistent standard error type 3; MBI-EE, Maslach Burnout Inventory–Emotional Exhaustion; MBL, mean sitting-bout length; MVPA, moderate-to-vigorous physical activity; pp, percentage points; PSQI, Pittsburgh Sleep Quality Index; PSS-10, 10-item Perceived Stress Scale; T1, baseline assessment; T2, follow-up assessment; WHO-5, World Health Organization–Five Well-Being Index.

For ΔMBL, expressed per 1-min/bout change, the associations with WHO-5 and MBI-EE were essentially unchanged after additional adjustment for baseline sedentary exposures. For WHO-5, the coefficient was *β* = −0.233 (95% CI − 0.437 to −0.029; *p* = 0.025) in Model 1 and *β* = −0.236 (95% CI − 0.441 to −0.032; *p* = 0.023; standardized *β* = −0.040; semi-partial *f*^2^ = 0.0071) in Model 2. For MBI-EE, the corresponding estimates were *β* = 0.127 (95% CI 0.036 to 0.217; *p* = 0.006) and *β* = 0.125 (95% CI 0.034 to 0.217; *p* = 0.007; standardized *β* = 0.041; semi-partial *f*^2^ = 0.0100), respectively. The association with PSS-10 was not statistically significant in either Model 1 [*β* = 0.070, 95% CI − 0.018 to 0.158; *p* = 0.120] or Model 2 [*β* = 0.068, 95% CI − 0.020 to 0.157; *p* = 0.131; standardized *β* = 0.029; semi-partial *f*^2^ = 0.0029]. Because Δ was defined as T2 − T1, these coefficient directions indicate that greater reductions in MBL were associated with higher WHO-5 and lower MBI-EE scores. For context, an interquartile-range reduction in MBL (4.11 min/bout) corresponded in Model 2 to an estimated 0.97-point higher WHO-5 score (95% CI 0.13 to 1.81), a 0.52-point lower MBI-EE score (95% CI 0.14 to 0.89 lower), and a 0.28-point lower PSS-10 score (95% CI 0.64 lower to 0.08 higher) (Table 2).

For ΔProlonged30%, expressed per 1-percentage-point change, the association with PSS-10 remained statistically significant after additional adjustment for baseline sedentary exposures. In Model 1, the coefficient was *β* = 0.041 (95% CI 0.004 to 0.077; *p* = 0.030), and in Model 2 it was *β* = 0.043 (95% CI 0.006 to 0.079; *p* = 0.022; standardized *β* = 0.045; semi-partial *f*^2^ = 0.0070). Thus, greater reductions in Prolonged30% were associated with lower PSS-10 scores at follow-up. In contrast, ΔProlonged30% was not associated with WHO-5 in either Model 1 [*β* = 0.005, 95% CI − 0.076 to 0.086; *p* = 0.899] or Model 2 [*β* = 0.008, 95% CI − 0.074 to 0.090; *p* = 0.846; standardized *β* = 0.003; semi-partial *f*^2^ = 0.0001], nor with MBI-EE in Model 1 [*β* = 0.017, 95% CI − 0.019 to 0.054; *p* = 0.355] or Model 2 [*β* = 0.020, 95% CI − 0.017 to 0.057; *p* = 0.284; standardized *β* = 0.016; semi-partial *f*^2^ = 0.0016]. For context, an interquartile-range reduction in Prolonged30% (10.20 percentage points) corresponded in Model 2 to an estimated 0.44-point lower PSS-10 score (95% CI 0.06 to 0.81 lower), whereas the corresponding estimated changes were 0.08 points lower in WHO-5 (95% CI 0.92 lower to 0.75 higher) and 0.21 points lower in MBI-EE (95% CI 0.58 lower to 0.17 higher) (Table 2).

For Δtotal sitting time, coefficients were scaled to a 30-min/day change for interpretability. The estimates were also essentially unchanged after adjustment for baseline sedentary exposures. For WHO-5, each 30-min/day greater increase in total sitting time from T1 to T2 was associated with a 0.529-point lower score in Model 1 (*β* = −0.529, 95% CI − 0.883 to −0.175; *p* = 0.003) and a 0.532-point lower score in Model 2 (*β* = −0.532, 95% CI − 0.888 to −0.177; *p* = 0.003; standardized *β* = −0.052; semi-partial *f*^2^ = 0.0120). For PSS-10, the corresponding coefficients were *β* = 0.223 (95% CI 0.067 to 0.378; *p* = 0.005) in Model 1 and *β* = 0.223 (95% CI 0.066 to 0.379; *p* = 0.005; standardized *β* = 0.056; semi-partial *f*^2^ = 0.0105) in Model 2. For MBI-EE, the estimates were *β* = 0.254 (95% CI 0.096 to 0.411; *p* = 0.002) and *β* = 0.255 (95% CI 0.098 to 0.413; *p* = 0.001; standardized *β* = 0.048; semi-partial *f*^2^ = 0.0138), respectively. Because Δtotal sitting time was defined as T2 − T1, these coefficient directions indicate that reductions in total sitting time were associated with higher WHO-5 and lower PSS-10 and MBI-EE scores (Table 2).

### 3.3 Correlation and multicollinearity diagnostics

Pairwise correlations among ΔMBL, ΔProlonged30%, Δtotal sitting time, and ΔMVPA were small, with absolute correlation coefficients ranging from 0.004 to 0.068 (Supplementary Table S1). The largest absolute correlation was observed between ΔMBL and Δtotal sitting time (*r* = −0.068, *p* = 0.059). Correlations between each baseline sedentary exposure and its corresponding change score were also very small: T1 MBL with ΔMBL (*r* = −0.002, *p* = 0.945), T1 Prolonged30% with ΔProlonged30% (*r* = −0.038, *p* = 0.295), and T1 total sitting time with Δtotal sitting time (*r* = 0.016, *p* = 0.664) (Supplementary Table S4). Multicollinearity diagnostics re-derived from the full fitted models are presented in Supplementary Table S2. Maximum VIFs remained low, ranging from 1.015 to 1.028 across the six models. In contrast, maximum condition indices derived from the full fitted design matrices ranged from 23.805 to 24.649 in Model 1 and from 38.299 to 39.171 in Model 2. Thus, inclusion of the baseline sedentary-exposure terms increased the condition indices of the full design matrices, although predictor-specific VIFs remained close to 1. This divergence can occur because VIF evaluates collinearity one predictor at a time through auxiliary regressions, whereas the condition index reflects near-dependencies in the entire scaled design matrix, including the intercept. Because the condition indices were derived from columns that were scaled but not mean-centered, they can also reflect alignment between the intercept and predictors with nonzero means. Accordingly, the higher condition indices do not contradict the low predictor-specific VIFs and were interpreted together with the stability of the focal coefficients and their HC3 standard errors. Importantly, re-examination of the Model 1 and Model 2 estimates showed that addition of the baseline exposure terms produced little change in the focal change-score coefficients or their HC3 standard errors, with no material attenuation or reversal of the primary associations.

### 3.4 Alternative parameterization using breaks in sitting

In this alternative parameterization, MBL was replaced by breaks in sitting per day while baseline and change terms for Prolonged30% and total sitting time were retained. Because total sitting time was retained in these models, the breaks/day specification represents an algebraic reparameterization of the underlying sitting-accumulation structure rather than an independent test of the MBL association. A greater increase in breaks/day was associated with higher WHO-5 scores (*β* = 0.223, 95% CI 0.120 to 0.326; *p* < 0.001; standardized *β* = 0.084; semi-partial *f*^2^ = 0.0230) and lower MBI-EE scores (*β* = −0.094, 95% CI − 0.143 to −0.046; *p* < 0.001; standardized *β* = −0.069; semi-partial *f*^2^ = 0.0205). The association with PSS-10 was not statistically significant (*β* = −0.034, 95% CI − 0.082 to 0.014; *p* = 0.169; standardized *β* = −0.033; semi-partial *f*^2^ = 0.0026). For interpretability, an increase of 5 breaks/day corresponded to an estimated 1.12-point higher WHO-5 score (95% CI 0.60 to 1.63) and a 0.47-point lower MBI-EE score (95% CI 0.23 to 0.72 lower), whereas the corresponding change in PSS-10 was −0.17 points (95% CI − 0.41 to 0.07). An interquartile-range increase in breaks/day (8.68 breaks/day) corresponded to an estimated 1.94-point higher WHO-5 score (95% CI 1.04 to 2.83) and a 0.82-point lower MBI-EE score (95% CI 0.40 to 1.24 lower). Overall, the alternative breaks/day parameterization expressed the same underlying sitting-accumulation structure in interruption-frequency units and yielded associations with WHO-5 and MBI-EE in the corresponding directions (Supplementary Table S3). The high model *R*^2^ values in Supplementary Table S3 largely reflected the strong association between each T1 outcome and its corresponding T2 outcome and should not be interpreted as variance explained by Δbreaks/day; the incremental contribution of Δbreaks/day is represented by the reported semi-partial *f*^2^ values.

### 3.5 Exploratory effect modification by baseline stress

Exploratory effect-modification analyses were conducted to examine whether baseline perceived stress modified the association between ΔMBL and WHO-5 at follow-up. In the primary analysis, baseline PSS-10 was modeled continuously. The ΔMBL × baseline PSS-10 interaction was not statistically significant (*β* = −0.024, 95% CI − 0.058 to 0.011; *p* = 0.182; semi-partial *f*^2^ = 0.0023), providing no statistical evidence that the association between ΔMBL and WHO-5 differed according to baseline stress level. At the mean baseline PSS-10 level, the estimated ΔMBL slope was *β* = −0.236 (95% CI − 0.441 to −0.032; *p* = 0.024). The corresponding simple slopes were *β* = −0.094 (95% CI − 0.408 to 0.220; *p* = 0.558) at 1 SD below the mean and *β* = −0.379 (95% CI − 0.650 to −0.109; *p* = 0.006) at 1 SD above the mean. Although the point estimates became more negative with increasing baseline stress, this pattern should not be interpreted as evidence of effect modification because the continuous interaction term was not statistically significant.

As a sensitivity analysis, baseline PSS-10 was dichotomized at the sample-specific 75th percentile (PSS-10 ≥ 21 vs. <21). The main effect of the high-stress indicator was included in the model and was not statistically significant (*β* = −0.494, 95% CI − 1.834 to 0.845; *p* = 0.469). The ΔMBL × high-stress interaction was statistically significant (*β* = −0.446, 95% CI − 0.879 to −0.014; *p* = 0.043). However, given the null result from the primary continuous-moderator analysis and the sample-specific nature of the dichotomized threshold, this sensitivity result was interpreted cautiously as exploratory. Full estimates from the primary continuous-moderator analysis and the dichotomized sensitivity analysis are provided in Supplementary Table S5.

## 4 Discussion

In this two-wave cohort of full-time university teachers, changes in sedentary-behavior indicators were associated with mental-health outcomes at follow-up in mutually adjusted ANCOVA models. Importantly, the estimated associations were materially unchanged when T1 MBL, T1 Prolonged30%, and T1 total sitting time were added alongside their respective change terms, indicating that the observed change-score associations were not materially altered after accounting for baseline sedentary-exposure levels. In the baseline-exposure-adjusted specification (Model 2), greater reductions in MBL were associated with higher WHO-5 scores and lower MBI-EE scores, greater reductions in Prolonged30% were associated with lower PSS-10 scores, and reductions in total sitting time were favorably associated with all three outcomes (Table 2). Overall, these findings indicate that longitudinal changes in both sedentary volume and sedentary accumulation characteristics were associated with mental-health outcomes in this cohort, although the magnitude and practical importance of these associations require careful interpretation.

The magnitude of these associations should be distinguished from their statistical significance. For WHO-5, an interquartile-range reduction in MBL (4.11 min/bout) corresponded to an estimated 0.97-point higher score, approximately one-tenth of the ~10-point contextual reference value, while a 30-min/day reduction in total sitting time corresponded to an estimated 0.53-point higher score. Although the ~10-point value has often been cited as a potentially meaningful change on the 0–100 WHO-5 scale, recent reviews have noted that a universally established minimally important difference for the WHO-5 is lacking (Domenech et al., 2025). For PSS-10, an interquartile-range reduction in Prolonged30% (10.20 percentage points) corresponded to an estimated 0.44-point lower score, approximately 16–20%—or about one-fifth—of the 2.2–2.7-point contextual reference range, while a 30-min/day reduction in total sitting time corresponded to an estimated 0.22-point lower score. The latter estimate was also well below this reference range (Drachev et al., 2020). A validated minimally important difference has not been established for the MBI-EE (Alvarez et al., 2020); nevertheless, the estimated changes associated with an interquartile-range reduction in MBL and a 30-min/day reduction in total sitting time were only approximately 0.52 and 0.26 points, respectively. Across the focal predictors in the primary Model 2 analyses, semi-partial *f*^2^ values ranged from 0.0001 to 0.0138, all below Cohen’s 0.02 benchmark for a small effect. Because these reference values were derived from populations other than university teachers and are not universally established thresholds, they should be interpreted as contextual benchmarks rather than definitive individual-level cutoffs for the present cohort. Taken together, these numerical comparisons indicate that, although several associations were statistically detectable, their estimated incremental magnitudes were small and are unlikely to represent changes that would be readily perceptible or clinically important for an individual teacher. The findings may therefore be more appropriately interpreted as evidence of small longitudinal associations at the population level rather than as evidence of clinically meaningful individual-level effects.

Our findings are consistent with the broader sedentary-behavior literature, which increasingly distinguishes sedentary volume from sedentary accumulation pattern. Evidence syntheses indicate that sedentary behavior is associated with several adverse health outcomes, and that the context and pattern of sedentary time may matter in addition to total duration (Saunders et al., 2020). More recent accelerometer-based prospective evidence has further linked higher sedentary time with an increased risk of future cardiovascular disease (Ajufo et al., 2025). For mental health, recent accelerometer-based prospective evidence has linked higher sedentary time with an increased risk of incident depression (Yu et al., 2025). Current physical activity and sedentary-behavior guidelines also recommend reducing sedentary time and replacing it with physical activity of any intensity where possible (Bull et al., 2020). The present study extends this evidence to university teachers by showing that longitudinal changes in objectively assessed sedentary volume and accumulation characteristics were both associated with mental-health outcomes. Given the structural relationships among these metrics, the accumulation findings should be interpreted as reflecting related features of how sitting was accumulated rather than as effects fully independent of total sitting time.

Because MBL is calculated as total sitting time divided by the number of sitting bouts, its interpretation is inherently linked to sitting-bout and break frequency. Conditional on total sitting time, a lower MBL reflects a greater number of sitting bouts and, correspondingly, more frequent breaks in sitting. Accordingly, the alternative parameterization using Δbreaks/day expressed the same underlying sitting-accumulation structure in interruption-frequency units: greater increases in breaks/day were associated with higher WHO-5 scores and lower MBI-EE scores, whereas the association with PSS-10 was not statistically significant (Supplementary Table S3). This analysis should therefore be interpreted as an alternative representation of the MBL-based association rather than as independent corroboration. The MBL findings are consequently better interpreted as reflecting a more frequently interrupted pattern of sitting rather than as an effect of bout duration in isolation. One possible explanation is that more frequent sitting interruptions may create repeated opportunities for micro-recovery during cognitively demanding academic work. According to Job Demands–Resources theory, sustained job demands without sufficient recovery can contribute to exhaustion, whereas resources and recovery opportunities can buffer strain and support well-being (Bakker et al., 2023). Interrupting prolonged sitting may provide brief opportunities for postural change, attentional disengagement, and physiological recovery during teaching preparation, grading, meetings, and computer-based work. Recent real-world experimental evidence among university employees showed that brief 3-min sedentary breaks produced small but positive immediate effects on affective valence and energetic arousal, although effects on calmness and working memory were not significant (Giurgiu et al., 2024). This interpretation is supported by evidence that micro-breaks can improve vigor and reduce fatigue (Albulescu et al., 2022), and by work-recovery research showing that recovery experiences are closely related to employee well-being (Bennett et al., 2018). Therefore, a more frequently interrupted sedentary accumulation pattern may represent not only a physical-behavioral change but also repeated opportunities for brief recovery throughout the working day.

Exploratory effect-modification analyses did not provide statistical evidence that baseline perceived stress modified the association between ΔMBL and WHO-5 when PSS-10 was modeled continuously. Although the dichotomized sensitivity analysis using the sample-specific 75th-percentile threshold yielded a statistically significant interaction, this finding was not supported by the primary continuous-moderator analysis and should therefore be interpreted cautiously. From a practical perspective, the alternative breaks/day parameterization provides a more directly interpretable behavioral expression of the same underlying sitting-accumulation structure represented by MBL: within this parameterization, a sitting pattern characterized by more frequent interruptions was associated with higher well-being and lower emotional exhaustion. These estimates should not be interpreted as independent corroboration of the MBL associations. However, given the modest magnitude of the estimated associations, these findings should not be interpreted as evidence that increasing break frequency by the observed amounts would produce a clinically meaningful improvement in mental health for an individual teacher. Nor does the observational design establish that prescribing a specific break frequency would improve mental health. Rather, the findings support further testing of feasible sitting-interruption strategies in adequately powered workplace intervention studies. Such strategies may include brief standing or walking breaks between periods of desk-based work, sit–stand workstations, movement breaks between classes or meetings, and digital prompts. Recent workplace intervention evidence indicates that activity-permissive workstations combined with multicomponent support can reduce prolonged sitting, with reductions maintained at 12-month follow-up (Wipfli et al., 2024). Productivity-focused evidence also suggests that sitting-reduction interventions do not necessarily impair work performance (Peterman et al., 2019). Experimental and meta-analytic evidence further indicates that regularly interrupting prolonged sitting with brief activity breaks can improve acute cardiometabolic responses, with more frequent interruptions, such as every 30 min, generally showing greater benefits for glucose control (Duran et al., 2023; Yin et al., 2024).

This study has several strengths. First, sedentary behavior was assessed using a thigh-worn activPAL4 monitor, which is well suited for distinguishing sitting/lying, standing, and stepping postures. A recent systematic review supports the validity of activPAL monitors for posture classification (O'Brien et al., 2022), and methodological guidance emphasizes their value in free-living sedentary-behavior research among adults (Edwardson et al., 2017). Second, both waves were conducted in the same month and academic term window, reducing potential bias from seasonal variation and academic-calendar effects. Third, the analytic strategy explicitly addressed the structural relationships among sedentary-behavior metrics by jointly modeling their change terms, additionally adjusting for their corresponding baseline levels, and evaluating pairwise correlations, predictor-specific VIFs, and condition indices derived from the full fitted design matrices. Because the corrected condition indices were higher in Model 2, we also re-examined the focal change-score coefficients and their HC3 standard errors across model specifications; these estimates remained materially stable after addition of the baseline exposure terms. An alternative parameterization using breaks/day provided a more directly interpretable expression of the same underlying sitting-accumulation structure in terms of interruption frequency, rather than an independent corroborative analysis of the MBL findings. Finally, the models accounted for concurrent changes in MVPA and sleep quality, two behavioral factors that are closely related to mental health.

Several limitations should be acknowledged. First, participants were recruited from a single university in Zhengzhou, China, which may limit the generalizability of the findings to other institutional, regional, and cultural settings. Teacher well-being and burnout are influenced by multiple individual and organizational factors, including workload, institutional support, job resources, and broader working conditions (Dreer, 2023; Zhou et al., 2024). Future multicenter studies should include universities with varied administrative structures, academic disciplines, teaching loads, and promotion systems. Second, the two-wave observational design does not permit causal inference and provides limited insight into the temporal processes linking changes in sitting patterns to changes in mental health. Third, the sedentary accumulation metrics are structurally related rather than statistically independent constructs. MBL is derived from total sitting time and the number of sitting bouts, while Prolonged30% is derived from the same underlying bout-length distribution. Therefore, adjustment for these variables within the same regression model does not make them conceptually or mathematically independent. Although predictor-specific VIFs remained close to 1, condition indices derived from the full fitted design matrices were higher, particularly in Model 2. Nevertheless, the focal change-score coefficients and their HC3 standard errors remained materially stable after the baseline exposure terms were added. These diagnostics should therefore be interpreted as assessments of numerical properties of the fitted models rather than evidence that the sedentary-behavior metrics are structurally independent. We therefore interpreted the MBL results as reflecting a more frequently interrupted pattern of sitting and used breaks/day as an alternative, more directly interpretable parameterization of the same underlying sitting-accumulation structure, rather than as an independent validation of the MBL findings. Fourth, the mental-health outcomes were self-reported and may therefore have been susceptible to reporting bias. Fifth, although MVPA was included as a covariate, it was estimated from activPAL4-derived activity-intensity outputs rather than measured using a device optimized specifically for activity-intensity classification. In addition, BMI was measured only at baseline and was modeled as a time-invariant covariate; unmeasured changes in BMI during follow-up may therefore have contributed to residual confounding. Sixth, other potentially relevant factors—including teaching hours, research pressure, administrative responsibilities, income, family burden, mental health diagnoses, medication use, and major life events—were not measured and may also have contributed to residual confounding.

Finally, the complete-case analysis excluded participants who were lost to follow-up or who did not provide device data meeting the predefined wear-time criteria or complete questionnaire data. Selection bias is therefore possible if participants included in the final analysis differed systematically from those who were excluded. Although reminder messages were sent via WeChat during the monitoring period, a considerable number of participants still did not provide device data that met the predefined wear-time criteria. Future studies could incorporate real-time wear-time monitoring and provide additional monitoring days when insufficient wear is detected to improve data completeness and reduce device-related exclusions.

## 5 Conclusion

In this two-wave cohort of full-time university teachers, more favorable changes in sedentary accumulation patterns were associated with better mental-health outcomes over approximately 12 months. Reductions in MBL were associated with higher well-being and lower emotional exhaustion, whereas reductions in Prolonged30% were associated with lower perceived stress. Because MBL is structurally linked to total sitting time and sitting-bout frequency, the MBL findings should be interpreted as reflecting a more frequently interrupted pattern of sitting rather than an isolated effect of bout duration. An interquartile-range reduction in MBL corresponded to a 0.97-point higher WHO-5 score, approximately one-tenth of the ~10-point contextual reference value, whereas an interquartile-range reduction in Prolonged30% corresponded to a 0.44-point lower PSS-10 score, approximately 16–20% of the 2.2–2.7-point contextual reference range. Moreover, all semi-partial *f*^2^ values for the focal predictors in the primary Model 2 analyses were below Cohen’s 0.02 benchmark for a small effect. These findings should therefore be interpreted as modest population-level longitudinal associations rather than as evidence of substantial individual-level changes. Nevertheless, these findings should be interpreted cautiously given the single-university sample, the observational two-wave design, potential selection bias, and the possibility of residual confounding. Future multicenter longitudinal studies and workplace interventions are needed to determine whether micro-breaks, sit–stand alternation, and other sitting-pattern strategies can causally improve mental health in academic settings.

## Data Availability

The original contributions presented in the study are included in the article/Supplementary material, further inquiries can be directed to the corresponding author.
